# Supplementary material for: An Interesting Conversion Route of Mononuclear Zinc Complex to Zinc Mixed Carboxylate Coordination Polymer
Source: Molecules. 2023 Feb 21;28(5):2011. doi: 10.3390/molecules28052011 (PMC10004649; doi:10.3390/molecules28052011)
Supplement: Supplementary file 1 [file molecules-28-02011-s001.zip › molecules-2158357-supplementary.pdf]

An interesting conversion route from mononuclear zinc complex to zinc coordination polymer

Gina Vasile Scăețeanu , Constantin G. Daniliuc ,Rodica Olar and Mihaela Badea

Table S1. Crystal data and structure refinement for compound (**1a**).

**Empirical formula**	$C_{14}H_{12}N_2O_4Zn$
Formula weight	337.65
Temperature/K	293(2)
Crystal system	*orthorhombic*
Space group	$Pca2_1$
a/Å	10.19420(10)
b/Å	8.7005(2)
c/Å	31.1599(4)
Volume/Å^3	2763.72(8) Å^3
Z	8
ρ_{calc} /g/cm^3	1.623
F(000)	1376
Radiation	MoKα′
Reflections collected	4650
Goodness-of-fit on F^2	1.083
Largest diff. peak/hole / e Å^{-3}	-0.302, 0.454

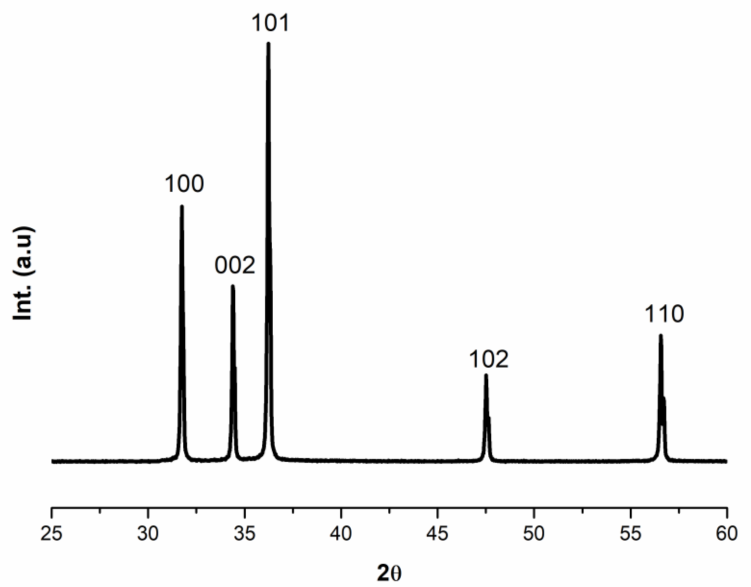

Figure S1. Powder X-ray diffractogram for final residue (ZnO) obtained from thermal decomposition of (**1a**).

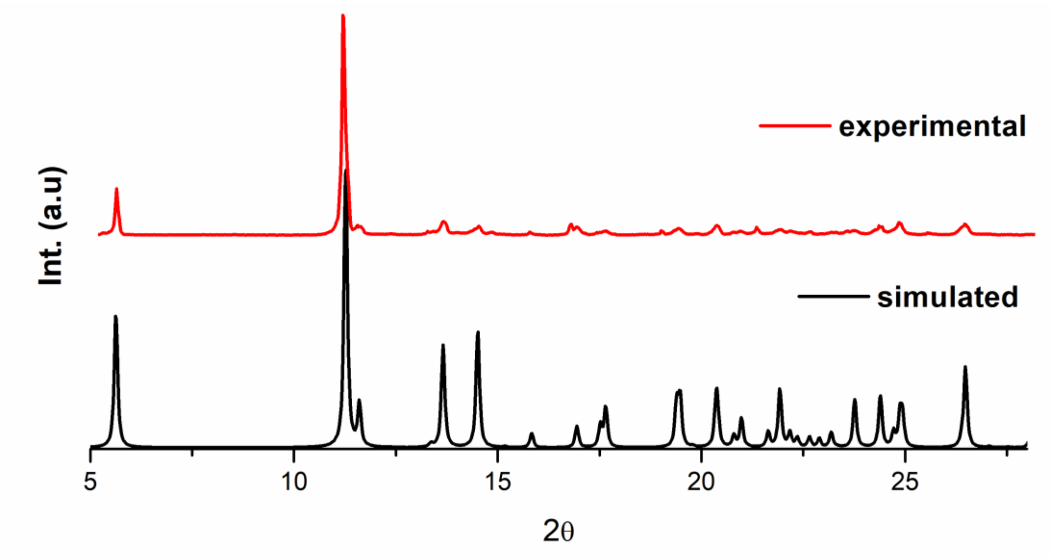

Figure S2. Powder X-ray diffractogram for complex (**1a**) (red) and the simulated one from single crystal X-ray data (black).
